# Influences of Elastic Foundations and Material Gradient on the Dynamic Response of Polymer Cylindrical Pipes Patterned by Carbon Nanotube Subjected to Moving Pressures

**DOI:** 10.3390/nano11113075

**Published:** 2021-11-15

**Authors:** Ali Deniz, Mahmure Avey, Nicholas Fantuzzi, Abdullah Sofiyev, Banu Esencan Turkaslan, Salim Yuce, Eckart Schnack

**Affiliations:** 1 Department of Mathematics, Faculty of Arts and Sciences, Usak University, Usak 64000, Turkey; ali.deniz@usak.edu.tr; 2 Division of Mathematics, Graduate Education Institute, Usak University, Usak 64000, Turkey; 2143052001@ogr.usak.edu.tr or; 3 Department of Civil, Chemical, Environmental and Materials Engineering, University Bologna, 40126 Bologna, Italy; nicholas.fantuzzi@unibo.it; 4 Department of Civil Engineering, Engineering Faculty, Suleyman Demirel University, Isparta 32260, Turkey; 5 Department of Chemical Engineering, Engineering Faculty, Suleyman Demirel University, Isparta 32260, Turkey; banuturkaslan@sdu.edu.tr; 6 Department of Mathematics, Faculty of Arts and Sciences, Yildiz Teknik University, Esenler Istanbul 34220, Turkey; sayuce@yildiz.edu.tr; 7 Department of Solids Mechanics, Karlsruhe Institute of Technology, 76131 Karlsruhe, Germany; eckart.schnack@kit.edu

**Keywords:** nanocomposites, polymer pipes, elastic foundations, moving pressures, CNTs, heterogeneity, dynamic coefficient, critical speed

## Abstract

Composite materials are frequently used in the construction of rail, tunnels, and pipelines as well as in the construction of aircraft, ships, and chemical pipelines. When such structural elements are formed from new-generation composites, such as CNT-reinforced composites, and their interaction with the ground, there is a need to renew the dynamic response calculations under moving pressures and to create new mathematical solution methods during their design. The aim of this study was to analyze the influences of elastic foundations (EFs) and material gradient on the dynamic response of infinitely long carbon nanotube (CNT)-based polymer pipes under combined static and moving pressures. The CNT-based polymer pipes resting on the EFs were exposed to the axial and moving pressures. The uniform and heterogeneous reinforcement distributions of CNTs, which varied linearly throughout the thickness of polymer pipes, were considered. After setting the problem, the fundamental equations derived to find new analytical expressions for dynamic coefficients and critical velocity, which are dynamic characteristics of cylindrical pipes reinforced by the uniform and linear distributions of CNTs, were solved in the framework of the vibration theory. Finally, numerical computations were performed to examine the effects of EFs on the critical parameters depending on the characteristics of the pipes, the speed of moving pressures, the shape of the distribution of CNTs, and the change in volume fractions.

## 1. Introduction

The simulation of the forced vibration of structural elements exposed to moving pressures is used in the design of rails, tunnels, and pipelines as well as in the design of missiles, aircraft, ships, and chemical pipelines [1,2]. Modeling the dynamic responses of homogeneous structural elements under moving pressures and methods for their solution were discussed from various aspects in the studies by Fryba [2]. The dynamic response of cylindrical pipes made of conventional homogeneous isotropic materials under moving pressures has been the subject of some research. For example, Forrestal and Alzheimer [3] studied the response of a circular elastic shell to moving and simultaneous loads. Huang [4] carried out a theoretical analysis of an axially symmetric, steady-state reaction of a linear-elastic, homogeneous, infinitely long cylindrical shell subjected to an annular moving pressure at a constant speed using the Fourier transform method with a contour integral. Singh et al. [5] investigated the dynamic axisymmetric response of buried orthotropic infinite cylindrical shells subjected to a radial line load moving along the shell’s axis within a thin shell theory. Panneton et al. [6] developed a theoretical model to evaluate the vibration and sound radiation of a thin cylindrical shell excited by a constant point load continuously traveling along the circumferential direction at a rotational speed. Ruzzene and Baz [7] developed a finite element model (FEM) to determine the critical velocities of axisymmetric shells subjected to axially moving loads. Karttunen and von Hertzen [8] studied the dynamic behavior of a homogeneous elastic cylinder cover using a Pasternak-type foundation model with Kelvin–Voigt damping. Eipakchi and coauthors [9,10] investigated the vibrational behavior of composite cylindrical shells with an auxetic honeycomb core layer and viscoelastic cylindrical shells subjected to a moving pressure. Sheng and Wang [11,12] presented coupling equations for controlling the electric potential and displacements of a functionally graded cylindrical shell with a surface-bonded piezoelectric layer and subject to thermal shock and moving loads using by Hamilton’s principle and Maxwell’s equation. Sofiyev and coauthors [13,14] examined dynamic response of an FGM cylindrical shell with and without elastic foundations under moving loads. Malekzadeh and Heydarpour [15] presented a transient thermoelastic analysis of functionally graded (FG) cylindrical shells under moving pressure and heat flux. Arazm et al. [16] presented an analytical procedure for the free vibration characteristics and dynamic response of an axisymmetric cylindrical shell that had a finite length, and it was made of functionally graded materials. Ramezani and Mirzaei [17] investigated the transient elastodynamic behavior of cylindrical tubes under moving pressures using different boundary conditions. Zhen et al. [18] presented analytical solutions to steady state responses of an infinite Euler–Bernoulli beam on a nonlinear viscoelastic foundation subjected to a harmonic moving pressure.

Since the first observations by Iijima nearly twenty years ago, carbon nanotubes have been the focus of important research [19]. In this process, many researchers have proved individually that carbon nanotubes have outstanding physical and mechanical properties. In addition to its unique electronic properties, high thermal conductivity, hardness, strength, and flexibility far superior to any existing material, carbon nanotubes offer tremendous opportunities for the development of fundamentally new material systems [20,21,22,23].

In connection with the development of technologies and extraordinary initiatives of material scientists, the production of new-generation heterogeneous composites and their wide participation in engineering applications as well as the creation of new calculation methods require a review of the calculations of cylindrical-shape structures originating from CNTs with and without EFs. For these reasons, the researchers have begun to study the static and dynamic responses of cylindrical shape structures originating from CNT. The first studies of the static and dynamic behavior of CNT-based cylindrical shells, without considering the influence of soils, were carried out, and there are many significant studies on this topic [24,25,26,27,28,29,30,31,32,33,34,35,36,37,38,39,40,41,42,43].

Currently, one of the most important tasks is to investigate the behavior of the forced vibration of new-generation heterogeneous structural elements reinforced with carbon nanotubes under moving pressure used in various media. Since the simultaneous influences of elastic media and heterogeneity on the dynamic coefficients and critical speed of new-generation composite structural elements can lead to unpredictable results, this issue should be considered in the design. The interaction of structural elements with elastic foundations has always been the focus of attention of the scientists. The simplest model for the elastic foundation is the Winkler model, which sees the foundation as a series of separated springs with no coupling effect among each other, causing the disadvantage of discontinuous deflection at the interacting surface of the structural elements. This was later improved by the introduction of a new dependent parameter by Pasternak [44], which considers the interactions between the separated springs in the Winkler elastic foundation model. Since then, the Pasternak elastic foundation model has been widely used to describe the mechanical behavior of structure–foundation interactions. One of the most realistic and widely used models among the foundation models is the Pasternak elastic foundation model. The basic information about other models of EFs and their interactions with structural elements is presented in detail in [45,46]. Since the dynamic problems of homogeneous cylindrical shells on the EFs were studied in early studies, a significant part of these studies was included in the books by Bazhenov [47].

In most applications of nanocomposites, the structural elements are embedded in an elastic environment or in contact with the EFs. This factor requires updating the investigation of the elastic foundation effects on the dynamic response of cylindrical-shape structures (in some cases infinitely long). The first attempt to study the effect of EFs on the dynamic behavior of cylindrical shells originating from CNTs was carried out in the study by Shen and Xiang [48]. After this study, starting in 2015, several studies on the dynamic and static problems of cylindrical shells originating from CNTs resting on the EFs were carried out, and their relevance and importance in applications are still being studied [42,49,50,51,52,53,54]. Despite the rapid increase in the number of studies devoted to the free vibration behavior of CNT-based cylindrical shells, panels, and pipes, the investigation on the forced vibration of such elements under moving pressures is scarce and has been conducted without considering the elastic foundations effect [55,56,57,58,59,60].

Despite the research effort devoted to the field, the investigation of the forced vibrations of cylindrical pipes based on CNTs of infinite length on elastic soils is still lacking. In previous studies, either the consideration of cylinders of finite length or obtaining solutions by numerical methods show that the analytical solution of the problem under consideration is very difficult from a mathematical point of view. In addition, the use of new-generation composites in structural elements interacting with elastic media, which have a wide range of applications in modern technology and are one of the first attempts to study the behavior of forced vibration under moving pressures, shows that the object under study is of actual and practical importance. The aim of the current investigation was to obtain the critical velocity as well as the dynamic deflections caused by moving pressures, compared with deflections caused by the static application of the same loads and linear distributions of CNTs in the matrix. Finally, a sensitivity analysis was performed using formulas containing the effect of EFs, and the influences of various parameters, such as the distribution shape of CNTs, volume fractions, and velocity of moving pressures on the forced vibration characteristics of infinite pipes were examined in detail compared to the unconstrained cylindrical pipes. The most important aspect of this study is that it is one of the first attempts to solve the forced vibration problem of polymer pipes reinforced by CNTs, taking into account the ground effect. The mathematical advantage of this work was to obtain a closed form solution for the critical velocity and dynamic coefficients. In addition, numerical analyses using analytical formulas are quantitatively and qualitatively unique. The advantage of this study for the engineer is that the results obtained can be used in different fields of engineering. For example, forced vibration simulation of CNT patterned structural elements subjected to moving pressure taking into account the ground effect can be considered during the design of missiles, aircraft, ships, and chemical pipelines as well as in the design of rails, tunnels, and pipelines.

## 2. Problem Description

**Figure 1:**
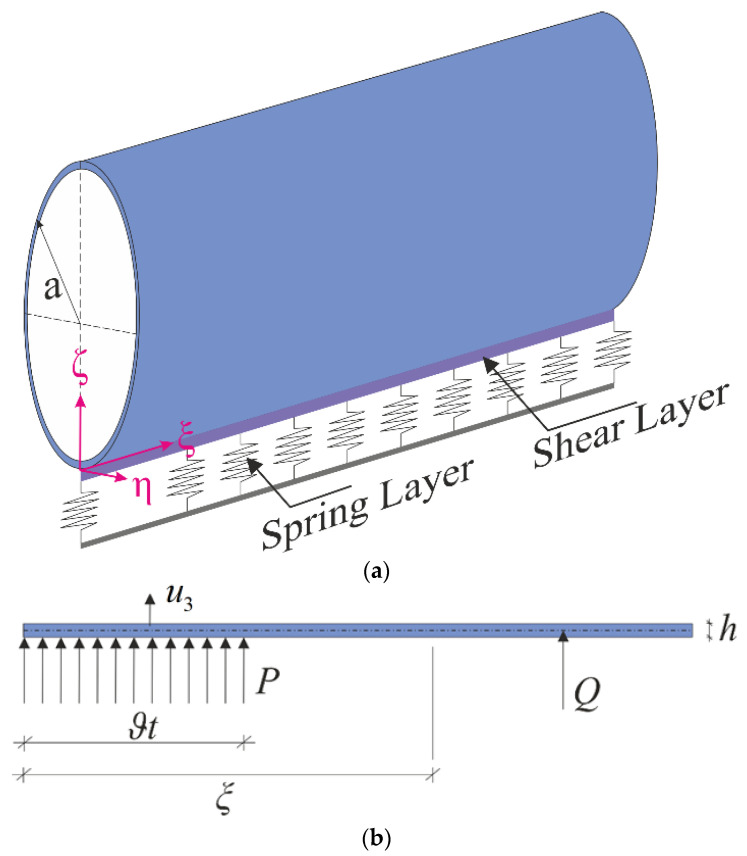
Scheme of an infinitely long cylindrical pipe (**a**) resting on the Pasternak elastic foundation (**b**) subjected to internal pressure P and annular pressure Q.

Consider an infinite cylindrical pipe originating from CNTs supported by elastic foundations with thickness h and radius a. The coordinate system (Oξηζ) and the coordinate axes were oriented as in Figure 1a. The reaction force ($N_{0}$) per unit area of the elastic foundation to the CNT-based pipe was formulated as follows [44,45,46,47]:

(1) $$N_{0}=k_{w}w-k_{p}\nabla^{2}w$$

where $k_{w}$ is the spring stiffness (in N/m^3^), $k_{p}$ is the shearing layer stiffness of the elastic foundation (in N/m), and $\nabla^{2}=\left(\frac{\partial^{2}}{\partial \xi^{2}}+\frac{\partial^{2}}{\partial \eta^{2}}\right)$ is the Laplace operator for ξ and η. Here, (0,0), $\left(k_{w},0\right)$, and $\left(k_{w},k_{p}\right)$ define the foundationless condition, the Winkler elastic foundation (WEF), and the Pasternak elastic foundation (PEF), respectively.

The CNT-based cylindrical pipe was under constant axial load T, internal pressure P, and ring-shaped moving pressure Q. Note that the annular pressure Q moves along the pipe from the left towards the right side of the cylinder with a constant velocity of ϑ, and the loading at the right-hand side of Q was assumed to be zero (Figure 1b).

### Modeling of Material Properties

The elasticity moduli and shear moduli, Poisson’s ratio, and density of polymer pipes and CNTs are indicated by $Y^{m},G^{m},\rho^{m},\nu^{m}$ and $Y_{i}^{CN},\;G_{12}^{CN},\;\rho^{CN},\;\nu_{12}^{CN}(i=1,2)$, respectively. By using the extended mixing rule [24], the effective mechanical properties of CNT-based polymer pipes are defined as follows:

(2) $$\begin{array}{ll} Y_{11}=\eta_{1}V_{CN}Y_{11}^{CN}+V^{m}Y^{m}, & \frac{\eta_{2}}{Y_{22}}=\frac{V^{CN}}{Y_{22}^{CN}}+\frac{V^{m}}{Y^{m}},\;\frac{\eta_{3}}{G_{12}}=\frac{V^{CN}}{G_{12}^{CN}}+\frac{V^{m}}{G^{m}}\\ G_{13}=G_{12},\;G_{23}=1.2\;G_{12}, & \nu_{12}=V_{*}^{CN}\nu_{12}^{CN}+V^{m}\nu^{m},\;\rho =V^{CN}\rho^{CN}+V^{m}\rho^{m} \end{array}$$

where $\eta_{1},\eta_{2}$ and $\eta_{3}$ are the denoted efficiency parameters, $V^{CN}$ and $V^{m}$ are the volume fractions of CNTs and the pipe and satisfies $V^{CN}=1-V^{m}$. Here $V_{*}^{CN}$ is described as [24]:

(3) $$V_{*}^{CN}=\frac{w^{CN}}{w^{CN}+(\rho^{CN}/\rho^{m})-(\rho^{CN}/\rho^{m})w^{CN}}\;$$

where $w^{CN}$ is the mass fraction of the nanotube.

**Figure 2:**
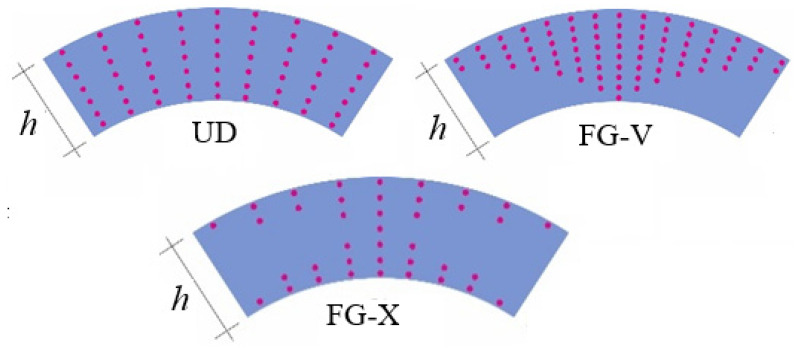
Different distribution patterns of CNTs: UD; FG-V; FG-X.

It was assumed that $V^{CN}$ is defined as a function of the thickness coordinate as follows [24,25,26,27,28,29,30,31,32,33,34,35,36,37,38,39,40,41,42,43] (see Figure 2):

(4) $$\begin{array}{lll} UD & \mathrm{at} & V^{CN}=V_{*}^{CN}\\ FG-V & \mathrm{at} & V^{CN}=(1-2\bar{\zeta })V_{*}^{CN},\;\bar{\zeta }=\zeta /h\\ FG-X & \mathrm{at} & V^{CN}=4\left|\bar{\zeta }\right|V_{*}^{CN} \end{array}$$

## 3. Basic Assumptions and Equations

For the axially symmetrical case of CNT-based polymer pipes, the forces and moments were defined as:

(5) $$\begin{array}{l} n_{11}=D_{1}\frac{\partial u_{1}}{\partial \xi }-D_{2}\frac{u_{3}}{a}-D_{4}\frac{\partial^{2}u_{3}}{\partial \xi^{2}},\;n_{22}=D_{2}\frac{\partial u_{1}}{\partial \xi }-D_{3}\frac{u_{3}}{a}-D_{5}\frac{\partial^{2}u_{3}}{\partial \xi^{2}},\\ m_{11}=D_{4}\frac{\partial u_{1}}{\partial \xi }-D_{5}\frac{u_{3}}{a}-D_{6}\frac{\partial^{2}u_{3}}{\partial \xi^{2}} \end{array}$$

where the following symbols are used:

(6a) $$\begin{array}{l} D_{1}=h\int_{-0.5}^{0.5}a_{11\bar{\zeta }}d\bar{\zeta },\;D_{2}=h\int_{-0.5}^{0.5}a_{12\bar{\zeta }}d\bar{\zeta }=h\int_{-0.5}^{0.5}a_{21\bar{\zeta }}d\bar{\zeta },\;D_{4}=h^{2}\int_{-0.5}^{0.5}\bar{\zeta }a_{11\bar{\zeta }}d\bar{\zeta },\\ D_{3}=h\int_{-0.5}^{0.5}a_{22\bar{\zeta }}d\bar{\zeta },\;D_{5}=h^{2}\int_{-0.5}^{0.5}\bar{\zeta }a_{12\bar{\zeta }}d\bar{\zeta },\;D_{6}=h^{3}\int_{-0.5}^{0.5}{\bar{\zeta }}^{2}a_{11\bar{\zeta }}d\bar{\zeta } \end{array}$$

in which

(6b) $$a_{11\bar{\zeta }}=\frac{Y_{11\bar{\zeta }}}{1-\nu_{12}\nu_{21}},\;a_{22\bar{\zeta }}=\frac{Y_{22\bar{\zeta }}}{1-\nu_{12}\nu_{21}},\;a_{12\bar{\zeta }}=\frac{\nu_{21}Y_{11\bar{\zeta }}}{1-\nu_{12}\nu_{21}}=\frac{\nu_{12}Y_{22\bar{\zeta }}}{1-\nu_{12}\nu_{21}}=a_{21\bar{\zeta }},\;a_{66\bar{\zeta }}=G_{12\bar{\zeta }}$$

Substituting relation (5), into the forced vibration equation for CNT-based polymer pipes subjected to axial load (*T*) and internal pressure (*P*) resting on the EFs, in the axisymmetric case, one gets:

(7) $$\delta_{1}\frac{\partial^{4}u_{3}}{\partial \xi^{4}}+\left(\delta_{4}+\frac{T}{2\pi a}-k_{p}\right)\frac{\partial^{2}u_{3}}{\partial \xi^{2}}+4\delta_{2}u_{3}=P-\frac{T}{2\pi a^{2}}\delta_{3}-\tilde{\rho }\frac{\partial^{2}u_{3}}{\partial t^{2}}$$

where

(8) $$\delta_{1}=\frac{D_{1}D_{6}-D_{4}^{2}}{D_{1}},\;\delta_{2}=\frac{D_{1}D_{3}-D_{2}^{2}}{4D_{1}a^{2}}+\frac{k_{w}}{4},\;\delta_{3}=\frac{D_{2}}{D_{1}},\;\delta_{4}=\frac{2\left(D_{1}D_{5}-D_{4}D_{2}\right)}{D_{1}a},\;\tilde{\rho }=h\int_{-0.5}^{0.5}\rho d\bar{\zeta }$$

Let us consider the new moving coordinate system for the ease of solving the problem. The point of application of the annular pressure Q was selected as the origin of the moving coordinate system, and the ξ axis is moving in the direction of x at speed ϑ [1,7,57].

Equation (7) can be expressed in the new coordinate system as follows:

(9) $$\delta_{1}\frac{d^{4}u_{3}}{dx^{4}}+\left(\bar{\rho }\vartheta^{2}+\delta_{4}-\frac{T}{2\pi a}-k_{p}\right)\frac{d^{2}u_{3}}{dx^{2}}+4\delta_{2}u_{3}=P-\delta_{4}\frac{T}{2\pi a^{2}}$$

The following transformations and symbols were used here [1,7,57]:

(10) $$\xi =x-\vartheta t,\;{\bar{u}}_{3}\left(x\right)=u_{3}\left(\xi -\vartheta t\right),\;\frac{\partial }{\partial \xi }=\frac{d}{dx},\;\frac{\partial }{\partial t}=-\vartheta \frac{d}{dx}$$

Since the first and fourth terms in Equation (9) are considerably larger than the second and third terms (see Appendix A), they can be ignored, and Equation (9) takes the form:

(11) $$\frac{\delta_{1}}{\delta_{2}}\frac{d^{4}{\bar{u}}_{3}}{dx^{4}}+\frac{\tilde{\rho }\vartheta^{2}-k_{p}}{\delta_{2}}\frac{d^{2}{\bar{u}}_{3}}{dx^{2}}+4{\bar{u}}_{3}-\left(P-\frac{T\delta_{3}}{2\pi a^{2}}\right)\frac{1}{\delta_{2}}=0$$

where $\delta_{1}/\delta_{2}$, which defines the stationary operating mode of CNT-based polymer pipes on EFs in the moving coordinate system, is the characteristic parameter of the strain change zone.

After the conversion of $x=\bar{x}{\left(\delta_{1}\delta_{2}{}^{-1}\right)}^{0.25}$ and ${\bar{u}}_{3st}=\left(P-\frac{T\delta_{3}}{2\pi a^{2}}\right)\frac{1}{4\delta_{2}}$ in Equation (11), one gets:

(12) $$\frac{d^{4}{\bar{u}}_{3}}{d{\bar{x}}^{4}}+4\mu \frac{d^{2}{\bar{u}}_{3}}{d{\bar{x}}^{2}}+4{\bar{u}}_{3}=4{\bar{u}}_{3st}$$

where

(13) $$\mu =\left(\tilde{\rho }\vartheta^{2}-k_{p}\right)\left(\delta_{1}\delta_{2}\right){}^{-0.5}$$

in which μ denotes the dynamic parameter, ${\bar{u}}_{3st}$ denotes the static displacement, and it is discontinuous at the cross-section ξ=0 due to the fact that the region is divided into area ξ<0 and ξ>0, then for ξ=0, we get ${\bar{u}}_{3}={\bar{u}}_{3st}$.

## 4. Solution Procedure

The general solution of (12) is sought as follows:

(14) $${\bar{u}}_{3}=Ce^{\lambda \bar{x}}$$

Introducing (14) into (12), the following characteristic equation is obtained:

(15) $$\lambda^{4}+4\mu \lambda^{2}+4=0$$

The roots of (14) are as follows:

(16) $$\lambda_{1,2}=b_{1}\pm ib_{2},\;\lambda_{3,4}=-b_{1}\pm ib_{2}$$

where

(17) $$b_{1}=\sqrt{1-\mu },\;b_{2}=\sqrt{1+\mu }$$

Since the cylindrical pipes originating from CNTs on the EFs have an infinite length, the strains cannot be infinitely increased with the increase in $\bar{x}$. Therefore, the solution for Equation (12) for the front and rear parts of moving pressure in the cylindrical pipe will be as follows:

(18) $${\bar{u}}_{3}={\bar{u}}_{3}^{left}+e^{b_{1}\bar{x}}\left[A_{1}\cos\left(b_{2}\bar{x}\right)+B_{1}\sin\left(b_{2}\bar{x}\right)\right]\;\mathrm{at}\;\bar{x}<0$$

and

(19) $${\bar{u}}_{3}={\bar{u}}_{3}^{right}+e^{-b_{1}\bar{x}}\left[A_{2}\cos\left(b_{2}\bar{x}\right)+B_{2}\sin\left(b_{2}\bar{x}\right)\right]\;\mathrm{at}\;\bar{x}>0$$

where $A_{j}\;and\;B_{j}\;(j=1,2)$ are unknown integral constants and are defined from the following assumptions.

The unknown integral constants are found under the following conditions. At the cross-section *ξ* = 0: (a) the deflection ${\bar{u}}_{3}$ has a rupture in the magnitude P, (b) the rotation angle and (c) the moment is continuous, and (d) the shear force has a rupture in the magnitude Q [1].

Algebraic equations are formed to determine $A_{j}\;and\;B_{j}\;(j=1,2)$, using the above assumptions as follows:

(20) $$\begin{array}{l} A_{1}-A_{2}=-\tilde{P}\\ b_{1}(A_{1}+A_{2})+b_{2}(B_{1}-B_{2})=0\\ (A_{1}-A_{2})\left(b_{1}^{2}-b_{2}^{2}\right)+2b_{1}b_{2}(B_{1}+B_{2})=0\\ (A_{1}+A_{2})\left(b_{1}^{2}-3b_{2}^{2}\right)b_{1}+(B_{1}-B_{2})\left(3b_{1}^{2}-b_{2}^{2}\right)b_{2}=-\tilde{Q} \end{array}$$

Solving the set of algebraic equations, one gets:

(21) $$\begin{array}{l} A_{1}=-0.5\tilde{P}+0.125{\left(1-\mu \right)}^{-0.5}\tilde{Q}\\ B_{1}=-\;0.5\mu {\left(1-\mu^{2}\right)}^{-0.5}\tilde{P}-0.125{\left(1-\mu \right)}^{-0.5}\tilde{Q}\\ A_{2}=0.5\tilde{P}+0.125{\left(1-\mu \right)}^{-0.5}\tilde{Q}\\ B_{2}=-\;0.5\mu {\left(1-\mu^{2}\right)}^{-0.5}\tilde{P}+0.125{\left(1-\mu \right)}^{-0.5}\tilde{Q} \end{array}$$

The critical velocity of the moving pressure for the cylindrical pipes originating from CNTs on the PEF is obtained by considering μ=1 into Equation (13):

(22) $$\vartheta_{cr}={\tilde{\rho }}^{-0.5}{\left[{\left(\delta_{1}\delta_{2}\right)}^{0.5}+k_{p}\right]}^{0.5}$$

The deflection ${\bar{u}}_{3}$ caused by the moving pressure in the range 0<μ<1 will be greater than the deflection at μ=0. Consequently, the dynamic coefficient of CNT-based polymer pipes on the EFs is defined as [1]:

(23) $$d_{F}={\bar{u}}_{3d}^{\max}/{\bar{u}}_{3st}^{\max}$$

where ${\bar{u}}_{3d}^{\max}={\left({\bar{u}}_{3}^{\max}\right)}_{\mu >0}$ and ${\bar{u}}_{3st}^{\max}={\left({\bar{u}}_{3}^{\max}\right)}_{\mu =0}$.

Moreover, ${\bar{u}}_{3}^{\max}$ is obtained from Equation (18). The ${\bar{x}}^{\max}$ is obtained from $\frac{d{\bar{u}}_{3}}{d\bar{x}}=0$:

(24) $$\tan(b_{1}{\bar{x}}^{\max})=\frac{2b_{2}\tilde{P}}{2b_{1}\tilde{P}-\tilde{Q}}$$

Now let us consider some special cases: (a) both maximum (dynamic and static) deflections for polymer pipes originating from CNTs on the PEF at P=0 and Q≠0 are defined as:

(25) $${\left({\bar{u}}_{3d}^{\max}\right)}_{Q\neq 0}=0.125\tilde{Q}{\left(1-\mu \right)}^{-0.5}\;\mathrm{and}\;{\left({\bar{u}}_{3st}^{\max}\right)}_{Q\neq 0}=0.125\tilde{Q}$$

The dynamic coefficient of the CNT-based cylindrical pipes on the PEF is found as follows:

(26) $$d_{F}^{Q\neq 0}={\left({\bar{u}}_{3d}^{\max}/{\bar{u}}_{3st}^{\max}\right)}_{Q\neq 0}={\left(1-\mu \right)}^{-0.5}$$

(b) Both the maximum (dynamic and static) deflections for cylindrical pipes originating from CNTs on the PEF at T= Q=0 and P≠0 are described as:

(27) $${\left({\bar{u}}_{3d}^{\max}\right)}_{P\neq 0}=\tilde{P}\left\{1+2^{-1.5}e^{-\Delta {\left[\left(1-\mu )/(1+\mu \right)\right]}^{0.5}}\left[{\left(1-\mu \right)}^{0.5}+\mu {\left(1-\mu \right)}^{-1}{\left(1+\mu \right)}^{-0.5}\right]\right\}$$

and

(28) $${\left({\bar{u}}_{3st}^{\max}\right)}_{P\neq 0}=\tilde{P}\left(1+2^{-1.5}e^{-\Delta_{0}}\right)$$

where

(29) $$0.5\pi \leq \Delta =-\arctan\left[{\left(1+\mu \right)}^{0.5}{\left(1-\mu \right)}^{-0.5}\right]+\pi \leq 0.75\pi =\Delta_{0}$$

Introducing Equations (27) and (28) into (23), the dynamic coefficient for CNT-based cylindrical pipes on the PEF takes the form:

(30) $$d_{F}^{P\neq 0}=\frac{2^{1.5}+e^{-\Delta {\left[\left(1-\mu )/(1+\mu \right)\right]}^{0.5}}\left[{\left(1-\mu \right)}^{0.5}+\mu {\left(1-\mu \right)}^{-1}{\left(1+\mu \right)}^{-0.5}\right]}{2^{1.5}+e^{-\Delta_{0}}}$$

In the expressions included in Formulas (22), (26), and (30), the corresponding expressions are obtained for cylindrical pipes originating from CNTs on the WEF when $k_{p}=0$ and unconstrained cylindrical pipes originating from CNTs when *k_w_* = *k_p_* = 0.

## 5. Numerical Examples

### 5.1. Comparisons

In order to confirm the accuracy of the obtained formulas, the first comparisons were conducted with the results of other studies, and the results are listed in Table 1 and Table 2.

In the first example, when the elastic ground effect was not taken into account, the critical velocity values of the infinitely long isotropic cylinder under the moving pressure were compared with the critical velocity values in the studies [1,7] and are listed in Table 1 using the Expression (22). In order to find the numerical values of the critical velocity, calculations were made by considering $E_{11}=E_{22}=E^{m}=210\;\mathrm{GPa},$ $G_{12}=G^{m}=78.948\;\mathrm{GPa},$ $\nu_{12}=\nu^{m}=0.33$, and $\rho =\rho^{m}=7800\;\mathrm{kg}/m^{3}$ in Formula (21). The variations in our numerical results between the values obtained in [1,7] for the critical velocity confirms the reliability of the obtained formula.

In the second example, ignoring the elastic ground effect, the values of the dynamic factors that occur in the presence of the internal (P≠0) and annular pressures (Q≠0) for an infinite-length isotropic cylinder were compared with the appropriate values presented in [1] and are listed in Table 2. To find the dynamic factors, namely, the magnitudes of $d_{F}^{Q\neq 0}$ and $d_{F}^{P\neq 0}$ in our study, Expressions (26) and (30) and the following data were used: h/a=0.2, $E_{11}=E_{22}=E^{m}=210\;\mathrm{GPa},$ $G_{12}=G^{m}=78.948\;\mathrm{GPa},$ $\nu_{12}=\nu^{m}=0.33$, $\rho =\rho^{m}=7800\;\mathrm{kg}/m^{3}$, and ϑ=1200 m/s. It can be seen that our results are in harmony with the results in [1].

### 5.2. Specific Numerical Results for the Dynamic Response of CNT-Based Cylindrical Pipes

In this subsection, the specific numerical results for the critical velocity and dynamic coefficients of CNT-based cylindrical pipes with different distributions and resting on the EFs discussed using the Maple 14. In addition, the influences of geometric parameters, material properties of composites reinforced by CNT, and the foundation characteristics of the critical velocity and dynamic coefficients were investigated.

In this study, the matrix was made from PMMA with the material properties of $E^{m}=2.5\;\mathrm{GPa}$, $\nu^{m}=0.34$, and $\rho^{m}=1150\;\mathrm{kg}/m^{3}$. The (10,10) armchair single-walled CNT was chosen as reinforcement for the tube with a length of 9.26 nm, radius of 0.68 nm, and thickness of s 0.067 nm. The elasticity moduli were $E_{11}^{CN}=5.6466\;\mathrm{TPa},$ $E_{22}^{CN}=7.08\;\mathrm{TPa}$ and $G_{12}^{CN}=1.9445\;\mathrm{TPa}$; the Poisson ratio was $\;\nu_{12}^{CN}=0.175$, and the density was $\;\rho^{CN}=1400\;\mathrm{kg}/m^{3}$ [48]. To clearly demonstrate the effects of CNTs on the critical parameters, three different volume fractions of the CNTs and the corresponding efficiency parameters $\eta_{j}(j=1,2,3)$ are presented in Table 3.

The variation in the critical velocity of moving pressure for polymer pipes originating from CNTs with an infinite length for different distributions and volume fractions versus *k_w_* and *k_p_* are given in Table 4. Here, (0,0), $\left(k_{w},0\right)$, and $\left(k_{w},k_{p}\right)$ define the foundationless condition, the WEF, and the PEF, respectively. As can be seen from Table 4, considering the effects of WEF and PEF, the critical velocity of the moving pressure acting on the cylindrical pipes of infinite length, reinforced by CNTs with the UD, FG-V, and FG-X types, was significantly reduced. The most obvious influence on the values of the critical speed occurred on the PEF compared to the WEF. In addition, considering the influence of the soil, it was found that the influences of the functionally graded distributions of CNTs over the cylinder thickness on the critical velocity were more pronounced than the foundationless condition. When the coefficients of EFs $k_{w}$ and $k_{p}$ increased, the influence of EFs on the critical speed became apparent, while an increase in $V_{*}^{CN}$ significantly reduced the influence of EFs on the critical velocity. In addition, when $V_{*}^{CN}$ increased, the effect of the functionally graded distributions of CNTs over the cylinder thickness on the critical velocity decreased, while a reduction in the inhomogeneity effect became slightly more pronounced, as the influences of WEF and PEF were taken into account. Since infinitely long FG-V and FG-X cylindrical pipes with and without EFs were compared with the infinitely long UD-cylinder with and without EFs, respectively, the influence on the critical speed was more noticeable for the FG-X cylinder. In addition, although the critical velocity of the UD-cylinder was greater than the critical velocity of the FG-V cylinder, it was less than the critical velocity of the FG-X cylinder. In the FG-X cylinder, as the coefficient $k_{p}$ was fixed and the coefficient $k_{w}$ changed, for example, as $\left(k_{w},k_{p})=(1\times 10^{9},\;1.5\times 10^{6}\right)$ and $\left(2\times 10^{9},\;1.5\times 10^{6}\right)$, the influences of the PEFs on the critical speed increased from +13.25% to +15.38%, from +12.45% to +12.95%, and from +13.52% to +14.25% for $V_{*}^{CN}$ = 0.12, 0.17, and 0.28, respectively. In the FG-X cylinder, if $k_{w}$ was fixed and $k_{p}$ changed, for example, as $\left(k_{w},k_{p})=(1\times 10^{9},\;0.5\times 10^{6}\right)$ and $\left(1\times 10^{9},\;1.5\times 10^{6}\right)$, the influences of PEFs on the critical speed increased from +11.52% to +13.25%, from +11.54% to +12.45%, and from +12.81% to +13.52% at $V_{*}^{CN}$ = 0.12, 0.17,s and 0.28, respectively. For all the values of the PEF coefficients presented in Table 4, the highest material gradient effect on the critical speed occurred at $V_{*}^{CN}$ = 0.12 at the FG-X cylinder. In addition, among the values of the coefficients $k_{w}$ and $k_{p}$ presented in Table 4, it can be seen that the change effect of the coefficient $k_{w}$ on the critical velocity was more pronounced than the change effect of the coefficient $k_{p}$.

**Figure 3:**
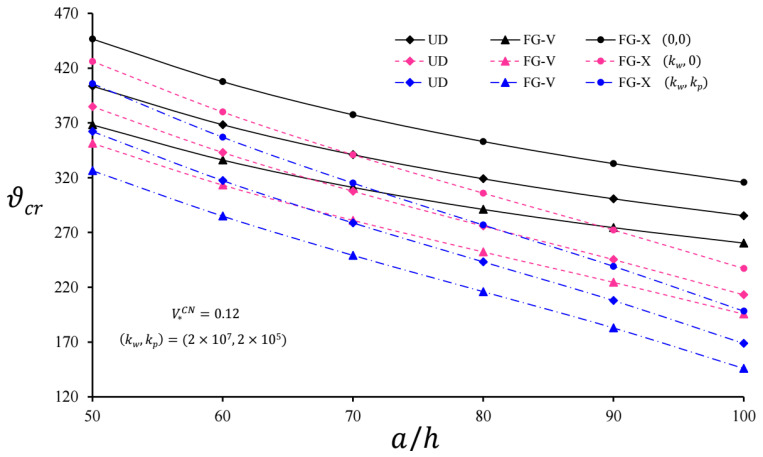
Variation in the critical velocity of infinite length CNT-based cylindrical pipes with and without EFs versus the *a*/*h* for $V_{*}^{CN}$ = 0.12.

The variation in the critical velocity of CNT-based polymer pipes with and without EFs versus the ratio *a*/*h* for $V_{*}^{CN}$ = 0.12 is plotted in Figure 3. With the increase in the *a*/*h* ratio, the critical velocity of the moving pressure affecting the cylindrical pipes originating from CNTs on WEF and PEF decreased faster than the unconstrained cylinders. At different values of the *a*/*h* ratio, the influence of the PEF on the critical velocity decreased more significantly than when considering the influence of the WEF. Although the effect of heterogeneity did not depend on the a/h in the unconstrained CNT-based cylindrical pipes, the effect of heterogeneity on the critical speed of the polymer pipes on the PEF became more pronounced, as the a/h increased for $V_{*}^{CN}$ = 0.12. For example, the effects of FG-V were −9.89% and −13.53%, while the effects of FG-X were +12.04% and +17.5%, respectively, as a/h = 50 and 100 for the composite cylinder on the PEF. The influence of both foundations on the critical speed increased due to the increase in a/h, and the influence of the PEF became apparent with the increase in a/h.

The variation of $d_{F}^{Q\neq 0}$ and $d_{F}^{P\neq 0}$ for the polymer pipes originating from CNTs versus $k_{w}$ and *k_p_* for ϑ=300 (m/s), a=30 h was calculated using Formulas (26) and (29) and is presented in Table 5. As can be seen from Table 5, the dynamic coefficients of the infinite length CNT-based cylindrical pipes with three different UD, FG-V, and FG-X types on the WEF and PEF increased with the increasing coefficients of shear and spring layers $k_{w}$ and $k_{p}$ together and separately.

The dynamic coefficients of CNT-based polymer pipes with all distribution shapes significantly decreased with the increasing in $V_{*}^{CN}$. The dynamic coefficient of the CNT-based polymer pipes with the shape FG-V on the EFs was higher than that of the pipe originating from the CNTs of the shape UD on the EFs, while the dynamic coefficient of the CNT-based polymer pipes with the shape FG-X on the EFs was lower. It was also found that cylindrical pipes originating from CNTs resting on EFs with the volume fraction $V_{*}^{CN}$ = 0.12 were more sensitive to internal and annular pressures than the CNT-based cylindrical pipes with other $V_{*}^{CN}$ (i.e., 0.17 and 0.28).

The use of EFs is one of the remarkable points about the heterogeneous distribution of CNTs that makes the influence of dynamic coefficients more obvious. An increase in $V_{*}^{CN}$ significantly reduced the influence of the inhomogeneous distribution of CNTs on the dynamic coefficient of polymer cylinders since both types of EF were taken into account. For example, at $\left(k_{w},k_{p}\right)$ = ($1.8\times 10^{8}$,$1.8\times 10^{5}$) and for the FG-V and FG-X cylinders, the greatest influence on the dynamic coefficient ($d_{F}^{P\neq 0}$) created by internal pressure was as follows: $V_{*}^{CN}$ = 0.17 were +% 1.62 and −% 1.24 and $V_{*}^{CN}$ = 0.28 were +0.77% and −0.87%, while these influences for $V_{*}^{CN}$ = 0.12 were observed as +11.29% and −5.77 %, respectively.

As the influences of the linear variation in the carbon nanotubes on $d_{F}^{Q\neq 0}$ and $d_{F}^{P\neq 0}$ were compared with each other, it was found that the influence of heterogeneity on $d_{F}^{Q\neq 0}$ was approximately twice that on the $d_{F}^{P\neq 0}$. As the coefficients $k_{w}$ and $k_{p}$ increased, both dynamic coefficients of the cylindrical pipes originating from CNTs became more pronounced compared to the unconstrained CNT-based cylindrical pipes. It was clear that due to an increase in $V_{*}^{CN}$, the influence of EFs on the $d_{F}^{Q\neq 0}$ and $d_{F}^{P\neq 0}$ for the CNT-based cylindrical pipes with infinite length was weakened.

**Figure 4:**
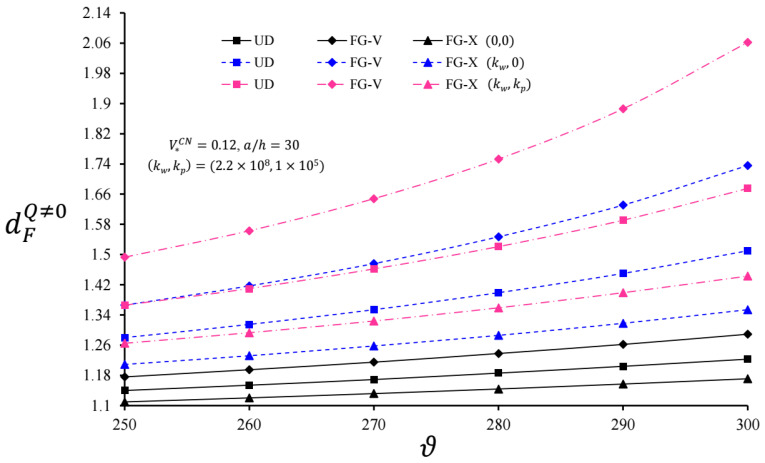
Variation in $d_{F}^{Q\neq 0}$ for infinite length CNT-based cylindrical pipes with and without EFs versus ϑ for $V_{*}^{CN}$ = 0.12 and a=30 h.

**Figure 5:**
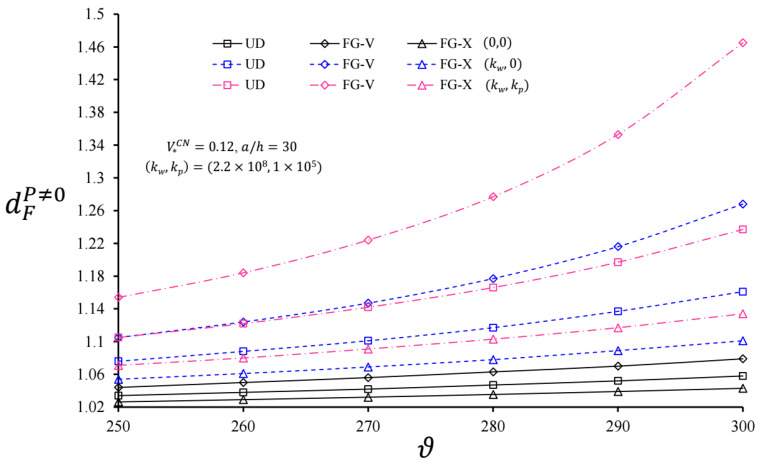
Variation in $d_{F}^{P\neq 0}$ for infinite length CNT-based cylindrical pipes with and without EFs versus ϑ for $V_{*}^{CN}$ = 0.12 and a=30 h.

The variation in $d_{F}^{Q\neq 0}$ and $d_{F}^{P\neq 0}$ for infinite length cylindrical pipes originating from CNTs with and without EFs versus ϑ for $V_{*}^{CN}$ = 0.12 and  a=30 h are plotted in Figure 4 and Figure 5, respectively. Here, (0,0), $\left(k_{w},0)=(2.2\times 10^{8},\;0\right)$, and $\left(k_{w},k_{p})=(2.2\times 10^{8},\;1\times 10^{5}\right)$ define the foundationless condition, the WEF, and the PEF, respectively. With the increase in ϑ, although the dynamic coefficients $d_{F}^{Q\neq 0}$ and $d_{F}^{P\neq 0}$ increased for the cylindrical pipes with and without EFs, the foundation effect accelerated the increase in $d_{F}^{Q\neq 0}$ and $d_{F}^{P\neq 0}$. It was observed that the heterogeneity effect was more prominent for the cylindrical pipes originating from CNTs on the EFs and increased with the increment of ϑ in comparison with UD-cylinders. For example, for the FG-V type cylinder, at $V_{*}^{CN}$ = 0.12 and $\left(k_{w},k_{p})=(2.2\times 10^{8},\;1\times 10^{5}\right)$, the FG-V effects on $d_{F}^{Q\neq 0}$ were +9.29% and +23.08%, whereas those on the $d_{F}^{P\neq 0}$ appeared to be +4.43% and +18.42%, as ϑ = 250 and 300 m/s, respectively. In the same conditions, the heterogeneity effect on the FG-X type cylinder was lower than the FG-V cylinder. In addition, the heterogeneity effects of the FG-V and FG-X cylindrical pipes on the WEF had a lower rate compared to the PEF.

## 6. Conclusions

In this study, the dynamic response of polymer pipes originating from CNTs resting on the EFs under moving pressures was investigated. We discussed the homogeneous and heterogeneous distributions of CNTs throughout the pipes’ thickness. The most important aspect of this study was that it is one of the first attempts to solve the forced vibration problem of polymer pipes reinforced with CNTs by considering the ground effect using vibration theory.

Numerical analyses supported the following generalizations:

(a) In the presence of the WEF and the PEF, the critical velocity values of endless length pipes reinforced with UD, FG-V, and FG-X type CNTs were significantly reduced;
(b) Considering the effect of soils, the influences of functionally graded distributions of CNTs over the cylinder thickness on the critical velocity were more pronounced than the foundationless condition;
(c) When the coefficients of the EFs increased, the influence of soils on the critical velocity became apparent, while the increase in $V_{*}^{CN}$ significantly reduced this effect;
(d) Since infinitely long FG-V and FG-X cylindrical pipes with and without EFs were compared with the infinitely long UD-cylinder with and without EFs, respectively, the influence on the critical speed was more noticeable for the FG-X cylinder;
(e) The critical velocity of moving pressure affecting the cylindrical pipes originating from CNTs on the WEF and the PEF decreased faster than the unconstrained cylinders with an increase in a/h;
(f) Although the effect of heterogeneity did not depend on the a/h in the unconstrained CNT-based cylindrical pipes, the effect of heterogeneity on the critical speed of the CNT-based polymer pipes on the PEF became more pronounced as the a/h increased;
(g) The dynamic coefficients of the infinite length CNT-based cylindrical pipes with UD, FG-V, and FG-X types on the WEF and PEF increased with the increasing coefficients of shear and spring layers together and separately;
(h) The dynamic coefficient of the CNT-based polymer pipes with the shape FG-V on the EFs was higher than that of the pipe originating from CNTs of the shape UD on the EFs, while the dynamic coefficient of the CNT-based polymer pipes with the shape FG-X on the EFs was lower;
(i) The use of EFs was one of the remarkable points and made the heterogeneous distribution of CNTs on the influence of the dynamic coefficients more obvious;
(j) An increase in $V_{*}^{CN}$ significantly reduced the influence of the inhomogeneous distribution of CNTs on the dynamic coefficient of polymer cylinders, since both types of EFs were taken into account;
(k) As the influences of the linear variation in carbon nanotubes on $d_{F}^{Q\neq 0}$ and $d_{F}^{P\neq 0}$ were compared with each other, the influence of heterogeneity on $d_{F}^{Q\neq 0}$ was approximately twice that on $d_{F}^{P\neq 0}$;
(l) With the increase in ϑ, although the dynamic coefficients increased for pipes with and without EFs, the foundation effect accelerated the increase of $d_{F}^{Q\neq 0}$ and $d_{F}^{P\neq 0}$;
(m) The heterogeneity effect was more prominent for cylindrical pipes originating from CNTs on the EFs and increased with the increment of ϑ in comparison with UD-cylinders.

Analysis and interpretations made using a closed form solution reveal that there are significant quantitative and qualitative changes in the forced vibration behavior of CNT-based polymer pipes. The criticalities revealed in the present work should be kept into account in the element design step in order to avoid accidents and damages that may occur in applications.

## Figures and Tables

**Table 1:** Comparisons of the critical velocity with the results of [1,7].

	[1]	[7]	Present Study
a/h	$\vartheta_{cr}$ (in m/s)		
10	1284 and 1265	1322	1284
50	565 and 574	591	574
100	400 and 406	418	406

**Table 2:** Comparisons of $d_{F}^{Q\neq 0}$ and $d_{F}^{P\neq 0}$ with the results in [1].

	[1]		Present Study	
μ	$d_{F}^{Q\neq 0}$	$d_{F}^{P\neq 0}$	$d_{F}^{Q\neq 0}$	$d_{F}^{P\neq 0}$
0.45	1.35	1.035	1.348	1.099

**Table 3:** The volume fractions of CNTs and corresponding efficiency parameters.

$V_{*}^{CN}$	0.12	0.17	0.28
$\eta_{1}$	0.137	0.142	0.141
$\eta_{2}$	1.022	1.626	1.585
$\eta_{3}$	0.715	1.138	1.109

**Table 4:** Variation in the critical velocity of the moving pressure for infinite length CNT-based cylindrical pipes versus $k_{w}$ and $k_{p}$ for a=10 h.

	$\vartheta_{cr}$			
$k_{w}(N/m^{3})$	$k_{p}(N/m)$	UD	FG-V	FG-X
	$V_{*}^{CN}$ = 0.12			
0	0	902.208	823.248	998.503
$1\times 10^{9}$	0	811.820	741.751	899.657
	$0.5\times 10^{6}$	785.289	712.616	875.791
	1.5 × 10^6^	729.337	650.443	825.992
2 × 10^9^	0	673.812	618.249	749.863
	0.5 × 10^6^	641.600	582.974	721.055
	1.5 × 10^6^	571.755	505.087	659.678
	$V_{*}^{CN}$ = 0.17			
0	0	1123.632	1025.740	1247.171
$1\times 10^{9}$	0	1061.269	969.987	1179.382
	$0.5\times 10^{6}$	1041.327	948.128	1161.470
	$1.5\times 10^{6}$	1000.253	902.823	1124.791
$2\times 10^{9}$	0	985.445	902.522	1097.353
	$0.5\times 10^{6}$	963.936	878.987	1078.079
	$1.5\times 10^{6}$	919.411	829.916	1038.459
	$V_{*}^{CN}$= 0.28			
0	0	1290.708	1187.119	1447.294
$1\times 10^{9}$	0	1227.587	1132.531	1380.741
	$0.5\times 10^{6}$	1210.779	1114.290	1365.820
	1.5 × 10^6^	1176.444	1076.882	1335.476
$2\times 10^{9}$	0	1152.882	1068.665	1302.878
	$0.5\times 10^{6}$	1134.969	1049.314	1287.054
	$1.5\times 10^{6}$	1098.265	1009.502	1254.807

**Table 5:** Variation in the dynamic coefficients of infinite length CNT-based cylindrical pipes versus the $k_{w}$ and $k_{p}$ (ϑ=300 m/s, a=30 h).

$V_{*}^{CN}=0.12$							
		UD		FG-V		FG-X	
$k_{w}$ (N/m^3^)	$k_{p}$ (N/m)	$d_{F}^{Q\neq 0}$	$d_{F}^{P\neq 0}$	$d_{F}^{Q\neq 0}$	$d_{F}^{P\neq 0}$	$d_{F}^{Q\neq 0}$	$d_{F}^{P\neq 0}$
0	0	1.223	1.058	1.289	1.079	1.171	1.043
$1.4\times 10^{8}$	0	1.337	1.095	1.455	1.139	1.249	1.066
	$1.2\times 10^{5}$	1.401	1.118	1.556	1.181	1.290	1.079
	$1.8\times 10^{5}$	1.437	1.132	1.616	1.208	1.312	1.087
$1.8\times 10^{8}$	0	1.413	1.123	1.572	1.188	1.296	1.081
	1.2 × 10^5^	1.500	1.157	1.722	1.261	1.349	1.099
	1.8 × 10^5^	1.549	1.178	1.815	1.311	1.378	1.110
$V_{*}^{CN}$ = 0.17							
0	0	1.128	1.031	1.160	1.040	1.100	1.024
$1.4\times 10^{8}$	0	1.153	1.038	1.193	1.049	1.119	1.029
	$1.2\times 10^{5}$	1.175	1.044	1.222	1.058	1.135	1.033
	1.8 × 10^5^	1.187	1.047	1.237	1.062	1.143	1.035
$1.8\times 10^{8}$	0	1.164	1.041	1.206	1.053	1.126	1.031
	$1.2\times 10^{5}$	1.187	1.048	1.238	1.063	1.143	1.035
	$1.8\times 10^{5}$	1.200	1.051	1.255	1.068	1.152	1.038
$V_{*}^{CN}$ = 0.28							
0	0	1.092	1.022	1.112	1.027	1.071	1.017
$1.4\times 10^{8}$	0	1.107	1.026	1.130	1.032	1.082	1.019
	$1.2\times 10^{5}$	1.121	1.029	1.147	1.036	1.092	1.022
	$1.8\times 10^{5}$	1.129	1.031	1.156	1.039	1.097	1.023
$1.8\times 10^{8}$	0	1.113	1.027	1.136	1.033	1.086	1.020
	$1.2\times 10^{5}$	1.128	1.031	1.155	1.038	1.097	1.023
	$1.8\times 10^{5}$	1.136	1.033	1.164	1.041	1.102	1.024

## Data Availability

Not applicable.

## Abbreviations

CNT	Carbon nanotube
EFs	Elastic foundations
FG-V	V-type functionally graded distribution
FG-X	X-type functionally graded distribution
PEF	Pasternak elastic foundation
WEF	Winkler elastic foundation
UD	Uniform distribution

## Appendix A

Comparing the first and the last terms of the left side of Equation (9), a characteristic parameter of the variation zone of the deformation is as follows:

(A1) $$d={\left(\frac{4\delta_{1}}{\delta_{2}}\right)}^{1/4}$$

Substituting Equation (A1) into Equation (9), it takes the following form:

(A2) $$\delta_{1}\frac{d^{4}u_{3}}{dx^{4}}+\left(\tilde{\rho }\vartheta^{2}+\delta_{4}-\frac{T}{2\pi a}-k_{p}\right)\frac{d^{2}u_{3}}{dx^{2}}+\frac{4\delta_{1}}{d^{4}}u_{3}=P-\delta_{4}\frac{T}{2\pi a^{2}}$$

Accordingly, in order of magnitude, Equation (A2) has the form:

(A3) $$\delta_{1}\frac{u_{3}}{d^{4}}+\left(\tilde{\rho }\vartheta^{2}+\delta_{4}-\frac{T}{2\pi a}-k_{p}\right)\frac{u_{3}}{d^{2}}+4\delta_{1}\frac{u_{3}}{d^{4}}\approx P$$

The rate of the second term to the first term in Equation (A3) is as follows:

(A4) $$\frac{\tilde{\rho }\vartheta^{2}\frac{u_{3}}{d^{2}}}{\delta_{1}\frac{u_{3}}{d^{4}}};\;\frac{\delta_{4}\frac{u_{3}}{d^{2}}}{\delta_{1}\frac{u_{3}}{d^{4}}};\;\frac{\frac{T}{2\pi a}\frac{u_{3}}{d^{2}}}{\delta_{1}\frac{u_{3}}{d^{4}}};\;\frac{k_{p}\frac{u_{3}}{d^{2}}}{\delta_{1}\frac{u_{3}}{d^{4}}}$$

It is clear that the second term in (A3) was much smaller than $\tilde{\rho }\vartheta^{2}$. It was proved that the third term was much smaller than $\tilde{\rho }\vartheta^{2}$ as follows:

(A5) $$\begin{array}{l} \frac{\frac{T}{2\pi a}\frac{u_{3}}{d^{2}}}{\delta_{1}\frac{u_{3}}{d^{4}}}=\frac{d^{2}}{\delta_{1}}\frac{T}{2\pi a}=\frac{d^{2}h}{\delta_{1}}\frac{T}{2\pi ah}={\left(\frac{4\delta_{1}}{\delta_{2}}\right)}^{1/2}\frac{h}{\delta_{1}}\frac{T}{2\pi ah}=\left[\frac{T}{2\pi ah}={\left(\sigma_{3}\right)}_{average}\right]\\ =\frac{2h{\left(\sigma_{3}\right)}_{average}}{\sqrt{\delta_{2}\delta_{1}}}<<\frac{2\tilde{\rho }\vartheta^{2}}{\sqrt{\delta_{2}\delta_{1}}}\Rightarrow h{\left(\sigma_{3}\right)}_{average}<<\tilde{\rho }\vartheta^{2} \end{array}$$

In addition, the fourth term ($k_{p}$) was not neglected as it was not much smaller than $\tilde{\rho }\vartheta^{2}$.
